# Genome-Wide Identification of Different Dormant *Medicago sativa* L. MicroRNAs in Response to Fall Dormancy

**DOI:** 10.1371/journal.pone.0114612

**Published:** 2014-12-04

**Authors:** Wenna Fan, Senhao Zhang, Hongqi Du, Xiaoge Sun, Yinghua Shi, Chengzhang Wang

**Affiliations:** College of Animal Science and Veterinary Medicine, Henan Agricultural University, Zhengzhou, China; Cankiri Karatekin University, Turkey

## Abstract

**Background:**

MicroRNAs (miRNAs) are a class of regulatory small RNAs (sRNAs) that regulate gene post-transcriptional expression in plants and animals. High-throughput sequencing technology is capable of identifying small RNAs in plant species. Alfalfa (*Medicago sativa* L.) is one of the most widely cultivated perennial forage legumes worldwide, and fall dormancy is an adaptive characteristic related to the biomass production and winter survival in alfalfa. Here, we applied high-throughput sRNA sequencing to identify some miRNAs that were responsive to fall dormancy in standard variety (Maverick and CUF101) of alfalfa.

**Results:**

Four sRNA libraries were generated and sequenced from alfalfa leaves in two typical varieties at distinct seasons. Through integrative analysis, we identified 51 novel miRNA candidates of 206 families. Additionally, we identified 28 miRNAs associated with fall dormancy in standard variety (Maverick and CUF101), including 20 known miRNAs and eight novel miRNAs. Both high-throughput sequencing and RT-qPCR confirmed that eight known miRNA members were up-regulated and six known miRNA members were down-regulated in response to fall dormancy in standard variety (Maverick and CUF101). Among the 51 novel miRNA candidates, five miRNAs were up-regulated and three miRNAs were down-regulated in response to fall dormancy in standard variety (Maverick and CUF101), and five of them were confirmed by Northern blot analysis.

**Conclusion:**

We identified 20 known miRNAs and eight new miRNA candidates that were responsive to fall dormancy in standard variety (Maverick and CUF101) by high-throughput sequencing of small RNAs from *Medicago sativa*. Our data provide a useful resource for investigating miRNA-mediated regulatory mechanisms of fall dormancy in alfalfa, and these findings are important for our understanding of the roles played by miRNAs in the response of plants to abiotic stress in general and fall dormancy in alfalfa.

## Introduction

There are two abundant classes of small RNAs (sRNAs) in plants; i.e., endogenous small interfering RNAs (siRNAs) and microRNAs (miRNAs) [1], [2]. Both siRNAs and miRNAs regulate messenger RNA (mRNA) stability and translation [1], and the miRNAs regulate gene expression by targeting specific mRNAs [2]. MiRNAs were firstly discovered as developmental timing regulators in *Caenorhabditis elegans* [3]. There is evidence that miRNAs play a critical role in post-transcriptional gene regulation and various biological and metabolic processes [4]. For instance, miRNAs regulate the modulation of the processes associated with growth and development in plants, including leaf morphogenesis, floral organs and root development [5]–[7]. Many new conserved Hg-responsive miRNAs and miRNA candidates were identified from *Medicago truncatula* [8]. Some of the miRNAs were involved in the regulation of development and plant responses to heavy metal stress; they were up- or down-regulated by heavy metals (Hg, Cd, and Al) [9]. In addition, miRNAs are involved in responses to various abiotic and biotic stresses, such as drought, cold, salinity and nutrient starvation [10]–[12].

Leguminous plants account for one third of primary crop production in the world [13]–[15]. In legumes, alfalfa (*Medicago sativa* L.) has been gradually concerned as an important forage crop in the world, due to its high nutrition value, wide adaptation, yield potential [16] and as a kind of nutritious feed. However, alfalfa varieties developed from different geographic regions have distinct characteristics in autumn. Ample studies have shown that there were high correlations between fall growth and winter injury in alfalfa [17]–[21], and the sugar content of roots and crown buds in alfalfa were closely related with winter hardiness [22]–[26]. Recent studies also revealed that winter injury was negatively associated with carbohydrate content in the roots of alfalfa, especially the hexosan content [27]. Increased sucrose, stachyose and raffinose, and reduced glucose, fructose and starch levels were related to freezing tolerance [24]. These studies mainly concerned the effects of temperature on fall dormancy of alfalfa. However, it has been reported that photoperiod was a key factor regulating fall dormancy in alfalfa [28]. Based on the responses of different varieties to low temperatures and short day-length (photoperiod), alfalfa varieties were classified into three types; i.e., fall dormant varieties grow very slowly and even cease to grow with short and decumbent shoots in the autumn. In contrast, non-dormant varieties continue to grow and produce tall upright shoots in the autumn. Semi-dormant varieties show phenotypes between dormant and non-dormant alfalfa [16], [29]. In many countries, such as the United States and Canada, FD class is commonly used as the first index of selecting alfalfa varieties because of its important role in various adaptations to particular regions associated with winter survival [30].

Recently, high-throughput sequencing analysis of sRNAs from *Medicago truncatula* leaves identified eight new miRNAs and sequencing sRNAs from specific tissues of legume plants by deep sequencing is expected to reveal more new miRNAs [31]. To identify the miRNAs in response to Hg, two small RNA libraries were generated from Hg-treated and Hg-free (control) *Medicago truncatula* seedlings. 201 individual miRNAs were identified representing 63 known *Medicago truncatula* miRNA families, including 12 new conserved and one non-conserved miRNAs that have not been described previously [8]. Zhou *et al.* used a bioinformatics approach for ESTs (Expressed Sequence Tags)- and GSS (Genomic Survey Sequences)-wide prediction of novel miRNAs in *Medicago truncatula* [9]. Lelandais *et al.* showed that spatial regulation of miRNAs may determine the specialization of regulatory RNA networks in plant differentiation processes in *Medicago truncatula*, such as root nodule formation [32].

Though the genome sequence of *Medicago truncatula* has been completed, which is an annual legume species with a small diploid genome and easy transformation and is a reference model plant to study functional genomics of alfalfa, the physiological, biochemical and molecular mechanisms causing FD are not clear. The genome sequence of alfalfa, which is an autotetraploid, allogamous and heterozygous species, is not available at present. Currently, we have sequenced the transcriptome of alfalfa using RNA-seq technology and identified some differentially expressed genes. Regulation of gene expression can be achieved at transcriptional, post-transcriptional and translational levels. In plants and animals, gene silencing occurs when endonuclease complexes are guided by small RNAs to target RNAs. For instance, a more effective approach to induce gene silencing in the alfalfa has been reported. Artificial microRNAs (amiRNAs) can be used to very specifically target genes for silencing because only a short sequence of 21 nucleotides of the gene of interest is used [33]. It is important to identify small RNAs and their target messenger RNAs, which are essential to understanding small RNA-mediated gene regulation of growth and stress responses and fall dormancy.

In comparison with miRNAs from *Medicago truncatula*, fewer miRNAs in *Medicago sativa* have been identified. In this study, we used previously known *Medicago truncatula* miRNAs to search for some miRNAs in response to fall dormancy of *Medicago sativa*. In this context, high-throughput sequencing has been used to identify alfalfa target miRNAs (including fall dormant and non-fall dormant types at two time points). To understand the role of miRNAs in response to fall dormancy of alfalfa, the objectives of this study were: (i) to sequence and annotate miRNAs of alfalfa varieties in different fall dormant types, and their expression in response to fall dormancy in standard variety (Maverick and CUF101), and (ii) to identify the target miRNAs related to fall dormancy in alfalfa and provide a scientific basis for miRNA function in fall dormancy of two standard alfalfa varieties (Maverick and CUF101).

## Materials and Methods

### Plant materials and growth condition

There are 11 fall dormancy classes (FDC) for *Medicago sativa* L. (alfalfa) at present, which are classified by the regrowth height after cutting [34] and can be broadly divided into dormant (FDC 1–4), semi-dormant (FDC 5–7) and non-dormant categories (FDC 8–11) [29]. In this study, alfalfa standard varieties Maverick (FDC1) and CUF101 (FDC9) were obtained from the United States and planted on a sandy loam soil at the Experimental Station of Henan Agricultural University, Zhengzhou, China (34°19×N, 113°35×E). The region experiences a monsoon-influenced, four-season humid subtropical climate with 640.8 mm of annual precipitation. Over the past 62 years, it has had a mean temperature of 14.7°C with extremes of −17.9°C and 43°C. Spring lasts from February 3 to May 4, summer from May 5 to August 6, autumn from August 7 to November 6, and winter from November 7 to February 2. Soil samples were collected and tested for amounts of nitrogen (N), phosphorous (P), and potassium (K). According to the results, the land was prepared by fertilizing 81 kg/ha of N as urea and 96 kg/ha with P_2_O_5_ as calcium phosphate to a depth of 15 cm. The field was in an alfalfa-alfalfa-alfalfa-corn (*Zea mays*)-winter wheat (*Triticum aestivum* L.) cropping system before the current study. On October 1, 2009, seed was sprinkled in 5 rows of alfalfa with 0.6 m row spacing plots. During drought, irrigation was provided in a timely manner. Weed control was performed by hand or hoeing, and insects were controlled as required.

Alfalfa leaves were collected from Maverick and CUF101, both on May 19 and September 23, 2011, which was 14 days after cutting. Samples of two different alfalfa varieties in May and September were named DM1 (Maverick, dormancy-May-1), NM2 (CUF101, non-dormancy-May-2), DS3 (Maverick, dormancy-September -3), NS4 (CUF101, non-dormancy- September-4), respectively. The two time points were selected because both the fall-dormant (Maverick) and non-fall dormant (CUF101) alfalfa grows normally in May, but in September, Maverick enters into fall dormancy while CUF101 still continues growing in our experimental station. Leaves were immediately frozen in liquid nitrogen and then stored at −80°C.

### RNA isolation and qualification

Total RNA was isolated using the TRIzol reagent (Invitrogen, Carlsbad, CA, USA) according to the manufacturer’s instructions. RNA degradation and contamination was monitored on 1% agarose gels. The RNA samples were measured with an ND 1000 spectrophotometer (Nanodrop) for contamination with either protein (A260 nm/A280 nm ratio) or reagent (A260 nm/A230 nm ratio). All the sample integrity numbers (RINs) were >7.5.

### Library construction and deep sequencing

Total RNAs were extracted from DM1, NM2, DS3 and NS4 following the manufacturer’s protocol. A total amount of 3 µg total RNA (≥100 ng/µl) per sample was used as input material to construct libraries. Sequencing libraries were generated using a NEB Next Multiplex Small RNA Library Prep Set for Illumina (NEB). Following manufacturer’s recommendations, index codes were added to attribute sequences to each sample. Briefly, NEB 3′ SR Adaptor was directly and specifically ligated to the 3′ end of miRNA. After the 3′ ligation reaction, we hybridized the SR RT primer. This step was important to prevent adaptor-dimer formation. For each sample, small RNAs (18–30 nt) were ligated with 5′ and 3′ RNA adapter by T4 RNA ligase, after they were purified by electrophoretic separation on a 15% TBE-urea denaturing PAGE gel. The sRNAs were transcribed into cDNA using Superscript II reverse transcriptase (Invitrogen, USA). Products were purified and quantified using the Agilent high sensitivity DNA assay on the Agilent Bioanalyzer 2100 system. After the clustering of the index-coded samples, four libraries, three for non-dormancy samples, one for dormancy sample, were sequenced on an Illumina Hiseq 2000 platform at the Beijing Novo Gene Genomics Institute, China.

### Analysis of sRNA sequencing data

Raw sequence reads were first processed through data cleaning by removing reads containing ploy-N, with 5′ primer contaminants, without 3′ primers or the insert tag, containing ploy A or T or G or C and low quality reads. At the same time, Q20, Q30, and GC contents of the raw data were calculated. Then, we chose a certain range of length from the clean reads to do all the downstream analyses. The available software miREvo and mirdeep2 were integrated to predict novel miRNA by exploring the secondary structure, the Dicer cleavage site and the minimum free energy of the small RNA tags unannotated in the former steps [35], [36].

### Mapping of sequence reads and expression analysis of miRNAs

Mapping of sequence reads back to the transcriptome was carried out using the *alignread.pl* script that comes with the Trinity assembler with the aligner bowtie (--bowtie) [37]. Sequences mapped to the genome using the *Medicago truncatula* (Mt3.0) used *SOAP* [38]. The expression levels of miRNA were estimated by transcript per million (TPM) through the following criteria Normalization formula: Normalized expression = mapped read count/Total reads×10^6^ [39]. P-values were adjusted using Q-value. Q-value<0.01 and |log_2_(fold change)|>1 was set as the threshold for significantly differential expression by default [40].

### Prediction and annotation of candidate potential miRNAs

Target accessibility allowed maximum energy to unpair the target-site (UPE). Target predictions were performed using the psRNA Target http://bioinfo3.noble.org/psRNA-Target/ and psRobot_v1.2 [41], through alignment with the genome of *Medicago truncatula* and the transcriptome of *Medicago sativa*, and then we took the intersection of the two predicting tools at the genome level of *Medicago truncatula* and transcriptome of *Medicago sativa*.

### Expression validation of different fall dormancy alfalfa miRNAs

This validation was done on five individual RNAs. We used SYBR Prime Script miRNA RT-PCR (TaKaRa, Code No.: RR716) for the RT reactions. All primers are listed in Table S1. The kit could add ploy (A) to the 3′ end of miRNAs and start reversely transcription. With known sequence at its 5′ end, the reverse transcription was led by special oligo-dT ligations. Real-time quantitative PCR was conducted on a light cycler Real-Time PCR Detection System. The reactions were incubated in a 96-well plate at 95°C for 300 s, followed by 40 cycles of 95°C for 10 s, 60°C for 10 s, and 72°C for 10 s. Each reaction included 1 µL of product from the diluted RT reactions, 0.8 µL of each forward primer, 0.8 µL of Uni-miR qPCR Primer, 10 µL of SYBR Premix Ex TaqII (TaKaRa, Code No.: RR716), and 7.4 µL of nuclease-free water. All reactions were run in three replicates for each sample. A relative quantitative method (2^−ΔΔCt^) was used to evaluate quantitative variation [42].

### Northern blot analysis

Northern blotting was performed as previously described [43]–[45]. Total RNA was isolated using the TRIzol reagent (Invitrogen, Carlsbad, CA, USA) according to the manufacturer’s instructions. About 30 µg of total RNA samples were run on 15% polyacrylamide denaturing (urea) gels, and then transferred to a Hybond-N+ nylon membrane (Amersham Biosciences) by electrophoresis using a semidry transfer cell (Bio-Rad). Hybridization was performed according to a standard protocol. Digoxin-labeled oligo-nucleotide probes complementary to the mature miR-novel_1, miR-novel_3, miR-novel_45, miR-novel_17 and miR-novel_76 were used in the hybridization. The probes are listed in Table 1.

**Table 1:** The probes designed for Northern blot.

Probes	Sequences of the probes
GAPDH	GCGCtaatacgactcactatagggTGGGAAGCACATTACAGCAG
novel_1	GCGCtaatacgactcactatagggCGGGATCGGAGATTAGAGAAT
novel_3	GCGCtaatacgactcactatagggTCGGACCAGGCTTCGTTCCCC
novel_45	GCGCtaatacgactcactatagggTCCGGTCCTTATTATAAGAAA
novel_17	GCGCtaatacgactcactatagggTTCGTCTTCTATATCTCTTTGG
novel_76	GCGCtaatacgactcactatagggGCAGCAGCATCAAGATTCACA

Note: All the nucleic acid sequences contain T7 promoter.

## Results

### Natural height and leaf area of alfalfa

Leaf areas were measured using A = KLW (A: leave area; K: coefficient of correction; L: Leave length; W: leave wide [46]). Twenty randomly selected plants were measured at each of the four conditions (alfalfa has a terrately compound leaf). Natural height (S Zhang, personal communication) and leaf areas are shown in Table 2. In Figure S1, DM1 (Maverick, May) and NM2 (CUF101, May) are shown to be growing well and similarly in May, while DS3 (Maverick, September) turned into a decumbent state and became much shorter than DM4 (CUF101, September) in September. From the plant height, Maverick might enter fall dormancy in September, while CUF101 did not show fall dormancy morphology at this time and continued to grow and produce tall upright shoots in the fall with a similar natural height and leaf area as in May and September [see Materials and methods].

**Table 2:** Natural height and leaf area of plants selected for sequencing.

Sample name	DM1	NM2	DS3	NS4
natural height(cm)	28.47±1.11	29.20±1.40	7.83±0.87	27.53±0.90
left-leaf(cm^2^)	1.78±0.29	1.72±0.32	1.44±0.34	1.75±0.36
middle-leaf(cm^2^)	2.01±0.33	2.02±0.39	1.66±0.37	2.09±0.41
right-leaf(cm^2^)	1.79±0.35	1.76±0.35	1.52±0.52	1.72±0.38

Plants heights and leaf areas were measured using Mean±SD (standard deviation).

### Analysis of sequences from four libraries

To investigate the enrichment of miRNAs and identify the miRNAs in response to fall dormancy, we generated four libraries from alfalfa leaves sampled under four conditions. After sequencing via Illumina Hiseq 2000, we removed low-quality reads and corrupted adapter sequences (reads<18 nt). High-throughput sequencing generated 7,536,934 reads (1,693,394 unique) representing DM1, 6,572,924 reads (1,985,915 unique) for NM2, 6,548,094 reads (1,449,168 unique) for DS3, and 6,627,260 reads (1,185,319 unique) for NS4. In order to control the quality of the libraries, we inspected the error rate and GC content of the sequencing results. They are presented in Table 3. The error rate distribution and GC distribution were also configured (ref. Figures S2 and S3).

**Table 3:** Basic characteristics of tags in four libraries.

Sample name	DM1	NM2	DS3	NS4
Raw reads	7,914,763	6,765,502	7,035,161	7,145,001
Clean reads	7,536,934	6,572,924	6,548,094	6,627,260
Unique Reads	1,693,394	1,985,915	1,449,168	1,185,319
Q20(%)	97.99	97.92	98.03	98.05
Q30(%)	94.43	94.46	94.34	94.70
GC content(%)	50.58	50.26	49.98	50.71

Note: the quality evaluation of original sample sequencing output data base on Q20, Q30, GC content.

**Figure 1:**
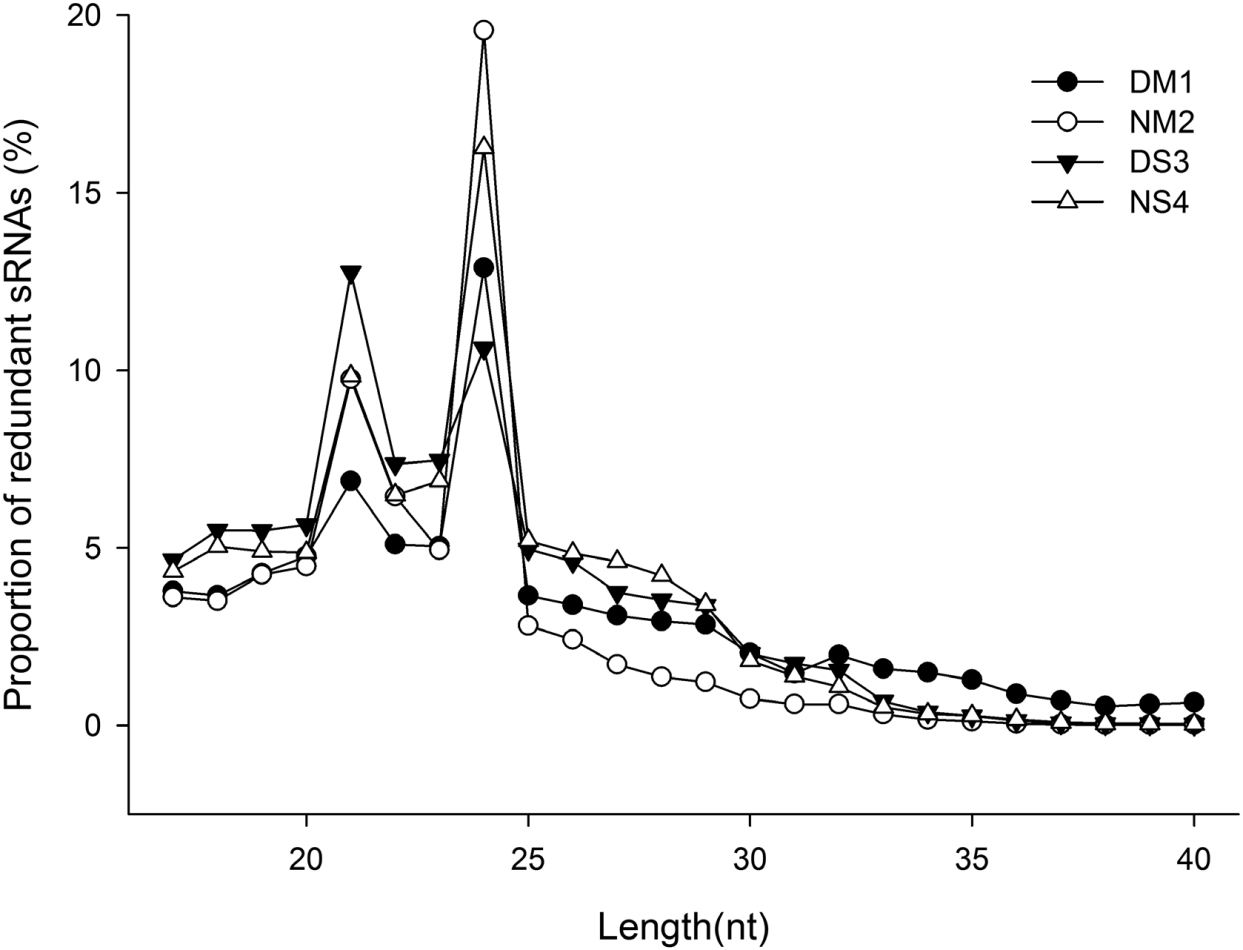
Distribution of sRNAs detected in four alfalfa libraries. The length distribution of sRNA reads in dormant alfalfa (DM1, and DS3) and non-dormant alfalfa (NM2, and NS4) were detected with Solexa technology.

The expression of miRNAs in dormant alfalfa (DM1 and DS3) and non-dormant alfalfa (NM2 and NS4) were examined with Solexa technology. The length distribution of reads showed that the majority of the reads, except for corrupted adapter sequences, were 20–25 nt in size (Figure 1). In the four libraries, 21 nt and 24 nt RNAs were the four most abundant classes (Figure 1). This was consistent with the distribution patterns of small RNAs from other plant species [32].

After initial processing, the high quality small RNA reads were mapped to the alfalfa transcriptome sequence (NCBI accession number: SRA057663) (S Zhang, personal communication). The number of total/unique sequences that matched the transcriptome were 2,745,473/358,615, 1,833,729/315,089, 2,963,552/341,796 and 2,012,514/248,641. The numbers of transcriptome-matched small RNAs with predicted hairpin structures were 4,710/566, 4,345/590, 4,931/460 and 2,535/455 in the four libraries, respectively (Table 4). The raw data have been submitted to Gene Expression Omnibus (GEO, http://www.ncbi.nlm.nih.gov/projects/geo/) and the NCBI accession number is SRP040470.

**Table 4:** Statistics of sRNA sequences for four alfalfa libraries.

Category	Number of reads	Unique sequence
**DM1**		
Total reads	6,029,847	1,693,394
Sequences mapped to the transcriptome	2,745,473	358,615
Match known miRNA	42,960	866
Transcriptome-matched small RNAs with predicted hairpins	4,710	566
**NM2**		
Total reads	5,680,282	1,985,915
Sequences mapped to the transcriptome	1,833,729	315,089
Match known miRNA	44,499	923
Transcriptome-matched small RNAs with predicted hairpins	4,345	590
**DS3**		
Total reads	5,178,165	1,449,168
Sequences mapped to the transcriptome	2,963,552	341,796
Match known miRNA	36,531	777
Transcriptome-matched small RNAs with predicted hairpins	4,931	460
**NS4**		
Total reads	3,703,470	1,185,319
Sequences mapped to the transcriptome	2,012,514	248,641
Match known miRNA	31,811	797
Transcriptome-matched small RNAs with predicted hairpins	2,535	455

Note: 1. Mapping of sequence reads back to transcriptome, using Trinity assembler with the aligner bowtie.

2. Screening sRNA within the scope of a certain length (15∼40 nt) for subsequent analysis (base on clean reads).

### Identification of known miRNAs and novel miRNAs in alfalfa

To identify known miRNAs in dormant and non-dormant alfalfa, we aligned the sRNA sequences with known mature miRNAs from plants in the miRBase20.0. Modified software mirdeep2 [36] and srna-tools-cli were used to obtain the potential miRNAs and draw the secondary structures. We identified 866, 923, 777 and 797 unique sequences as known miRNAs in the four alfalfa libraries (Table 4), respectively.

Novel miRNAs were predicted according to the characteristic hairpin structures of their precursors, which distinguishes them from other endogenous sRNAs [47], [48]. A total of 51 novel hairpin miRNAs candidates (206 families) were identified (Table S2, Table S3), which were not found in other species. These miRNA candidates are likely to be new miRNAs or new members of known miRNA families in alfalfa.

### Potential miRNAs related to fall dormancy in alfalfa

We took the intersection of the two predicting tools (psRNA Target and psRobot_v1.2) at the genome level of *Medicago truncatula* (Table S4) and the transcriptome of *Medicago sativa* (Table S5). We identified 566 potential miRNAs related to fall dormancy at the genome level of *Medicago truncatula*, and 583 potential miRNAs related to fall dormancy at the level of the transcriptome of *Medicago sativa*. Their predicted function annotation, target accession, target start, target end, expectation, UPE, target aligned fragment are listed in Table S4 at the genome level of *Medicago truncatula* and an Table S5 at the transcriptome level of *Medicago sativa*.

### Functional annotation of differentially expressed miRNAs

**Figure 2:**
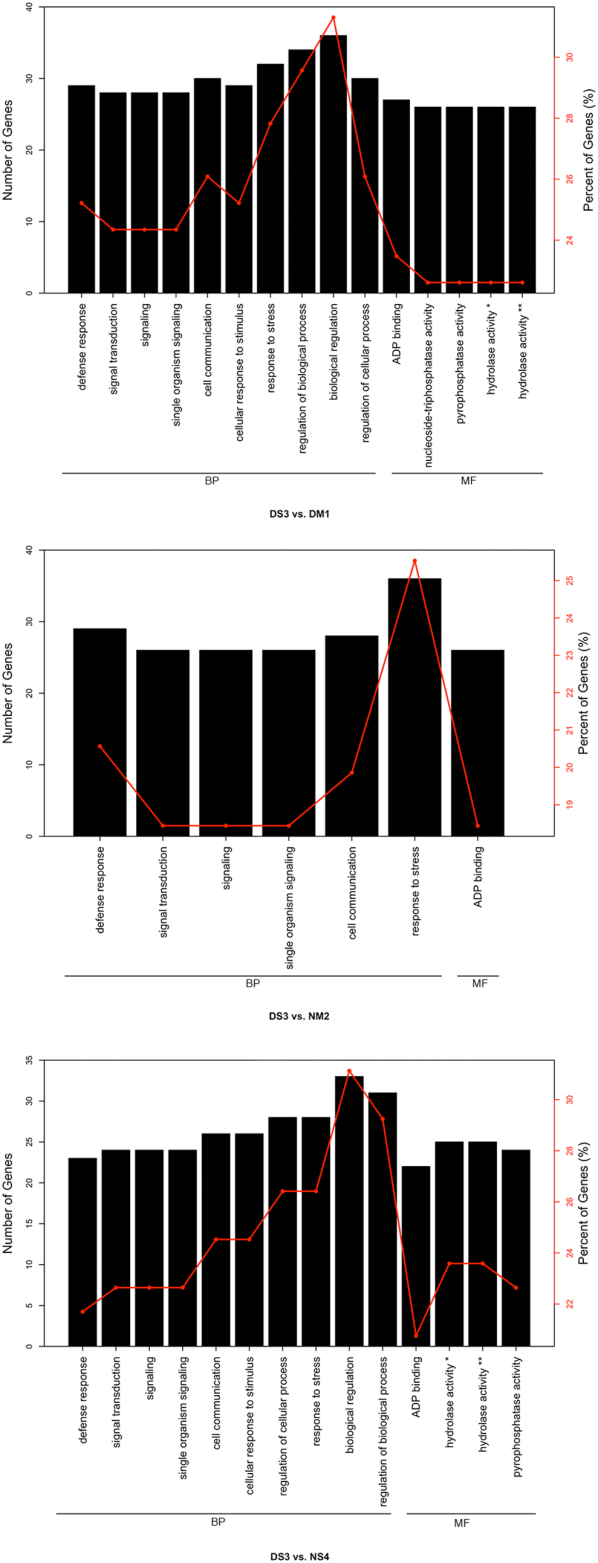
GO functional enrichment of genes differentially expressed in alfalfa. Gene classification based on gene ontology (GO) for the differential expression miRNA target genes in DS3 vs DM1, DS3 vs NM2 DS3, and DS3 vs NS4. The number of genes in GO terms was analyzed using GO Slim Assignment. Biological processes and molecular function were used for GO analysis. (Hydrolase activity* = hydrolase activity, acting on acid anhydrides, in phosphorus-containing anhydrides;hydrolase activity** = hydrolase activity, acting on acid anhydrides; BP, Biological Process; MF, Molecular Function).

Gene Ontology (GO) enrichment analysis was used on the target gene candidates of differentially expressed miRNAs [49]. GO functional enrichment analysis was performed to characterize the functional consequences of changes in gene expression associated with fall dormancy. According to the GO enrichment, the differential expression miRNA target genes in alfalfa related to defense responses, signal transduction, signaling, single organism signaling, cell communication, cellular responses to a stimulus, regulation of cellular processes, response to stress, biological regulation, regulation of biological processes, ADP binding, hydrolase activity, and pyrophosphatase activity (Figure 2). From Figure 2, we can see that fall dormancy is mainly a biological process in alfalfa. The main GO enriched factors are biological regulation, regulation of biological processes, response to stress, and regulation of cellular processes.

KEGG pathways were used to assess the statistical enrichment of the target gene candidates via KOBAS software. In three sets, the statistical enrichment of the gene candidates in KEGG pathways showed that the majority of target gene clusters included metabolic pathways, biosynthesis of secondary metabolites, porphyrin and chlorophyll metabolism, glycerolipid metabolism, cell cycle, glycerolipid metabolism, meiosis, plant hormone signal transduction, glycerophospholipid metabolism, ubiquitin-mediated proteolysis, etc. (Table S6). From the most enriched pathway terms, we can see that the main enriched factors included metabolic pathways, biosynthesis of secondary metabolites, cell cycle, and ubiquitin mediated proteolysis. From the analysis of GO enrichment and KEGG pathways, we inferred that the regulation mechanism of fall dormancy was biological regulation of responses to stress in the metabolic pathway.

### Response of known miRNAs to fall dormancy in different types of dormant alfalfa

**Figure 3:**
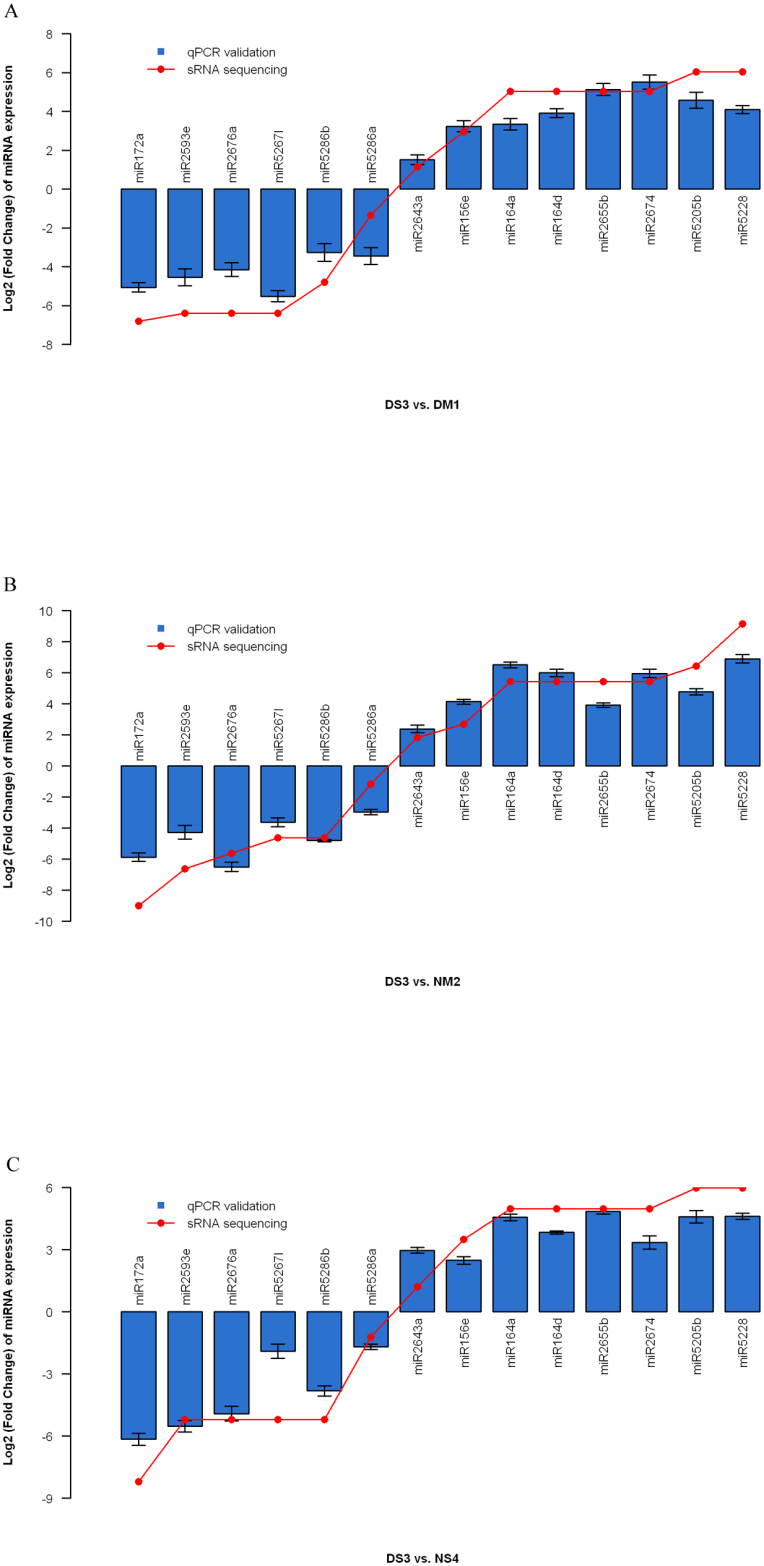
Sequencing and qRT-PCR. Quantitative RT-PCR validation of differentially expressed genes in non-dormant alfalfa (NM2, and NS4) and dormant alfalfa (DM1, and DS3), including 14 genes. All data were normalized to the expression level of GAPDH. Data represent Log_2_ (Fold Change) of relative quantifications for DM1, NM2, and NS4 vs DS3. The error bars represent the range of the fold change as determined by the data assist software.

To identify fall dormancy-responsive miRNAs in alfalfa, the normalized expression of miRNAs in the DS3 library was compared to that in the DM1, NM2 and NS4 libraries (Tables S7, 8, and 9). In contrast to DS3 vs DM1, DS3 vs NM2 and DS3 vs NS4, there were 11 up-regulated miRNAs and nine down-regulated miRNAs found in three sets (Figure S4). The differences in the expression profiles of these miRNAs indicated that each miRNA may play a different role in alfalfa, and they may explain the differences between dormant alfalfa and non-dormant alfalfa. Based on the results of high-throughput sequencing, we selected 14 miRNAs from these 20 known miRNAs randomly with changes in expression levels with Log_2_ (Fold Change)> = 1 (P value<0.01) [40] in response to fall dormancy treatment to validate the expression patterns with real-time quantitative PCR. As shown in Figure 3, high-throughput sequencing and RT-PCR produced similar results (Figure 3). Figure S3 shows miR172a, miR52671, miR2676a, miR2593e, miR5286b, miR5281a, miR2634, miR5286a, and miR2629h to be down-regulated in different types of dormant alfalfa. Conversely, miR5228, miR5205b, miR5279, miR2674, miR2655b, miR164a, miR164d, miR156g-3p, miR156e, miR2643a, and miR5299 were up-regulated in different types of dormant alfalfa. We picked up these common miRNAs from three contrasts; i.e., DS3 vs DM1, DS3 vs NM2 and DS3 vs NS4 (Figure S2), and gave their expression pattern and function annotations in Table 5. Because little is known about miRNA-mediated developmental regulation of fall dormancy in alfalfa, we can see that most function annotations of known miRNAs in response to fall dormancy in different types of dormant alfalfa are hypothetical proteins, as shown in Table 5.

**Table 5:** Predicted targets of fall dormancy miRNAs and their function annotations.

miRNAs	Expressionpattern	Functions annotations
miR5228	up	serine/threonine-protein phosphatase 7-like
miR5205b	up	hypothetical protein MTR_6g042030
miR5279	up	No hit
miR2674	up	Resistance protein [*Medicago truncatula*]
miR2655b	up	cleavage and polyadenylation specificity factor
miR164a	up	NAC domain protein contig_103035
miR164d	up	NAC domain protein chr2
miR156g-3p	up	UDP-N-acetylglucosamine–peptide
miR156e	up	Squamosa promoter-binding-like protein contig_52418
miR2643a	up	F-box/kelch-repeat protein chr5
miR5299	up	hypothetical protein chr4
miR172a	down	Transcription factor APETALA2
miR52671	down	GDSL esterase/lipase contig_9183
miR2676a	down	F-box/WD-40 repeat-containing protein
miR2593e	down	S-locus lectin protein kinase family protein
miR5286b	down	hypothetical protein chr8
miR5281a	down	hypothetical protein chr8
miR2634	down	Cys2/His2 zinc-finger transcription factor
miR5286a	down	hypothetical protein chr8
miR2629h	down	hypothetical protein chr1

### Response of new miRNAs to fall dormancy in different types of dormant alfalfa

**Figure 4:**
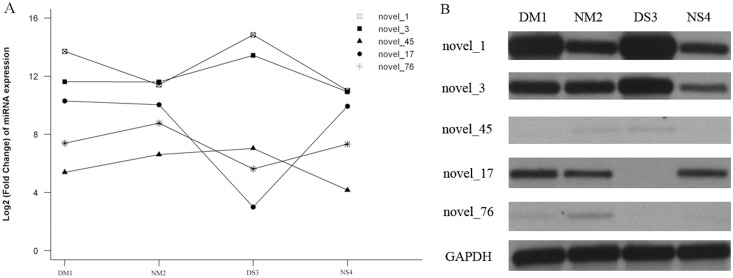
Northern blot analysis. Sequencing Data represent Log_2_ (Fold Change) of quantification of DM1, NM2, DS3, and NS4. Northern blot analysis showed the different expressions of miR-novel_1, miR-novel_3, miR-novel_45, miR-novel_17 and miR-novel_76 in non-dormant alfalfa (NM2, and NS4) and dormant alfalfa (DM1, and DS3). GAPDH was used as a control.

Among novel miRNAs, there were five candidate up-regulated miRNAs and three down-regulated miRNAs found in three sets (Table S10). It shows novel_17, novel_75, and novel_76 to be down-regulated in different dormant varieties. Conversely, novel_1, novel_3, novel_42, novel_45, and novel_85 were up-regulated in different types of dormant alfalfa. Based on the results of high-throughput sequencing (Figure 4A), we selected five novel miRNAs from these eight novel miRNA candidates randomly. The five novel miRNA levels were further confirmed by Northern blotting analysis (Figure 4B). As shown in Figure 4, high-throughput sequencing and Northern blotting analysis of the five novel miRNAs produced similar results (Figure 4A, and 4B). The increasing miR-novel_1, miR-novel_3, and miR-novel_42 levels were confirmed by Northern blotting analysis. At the same time, the decreasing miR-novel_17 and miR-novel_76 levels also had a similar variation trend with sequencing. The miRNA cluster analysis of differentially expressed sRNAs is shown in Figure S5. It was performed based on fold-changes between DM1, NM2, DS3, and NS4. Cluster analysis of differentially expressed sRNA in different types of dormant alfalfa was displayed using different colors. One hundred and forty six miRNAs (known miRNAs and novel miRNAs candidates) were potentially involved in the regulation of fall dormancy, as shown in Figure S5.

## Discussion

For most eukaryotic cells, miRNAs are a class of regulatory sRNAs involved in gene regulation. Recent studies have shown that miRNAs play an important role in diverse abiotic and biotic stresses, including drought, cold, nutrient starvation, oxidative stress, submergence, UV-B radiation, and viruses [5]–[7], [10], [39], [50], [51]. *Medicago truncatula*, which is an annual legume species and a model plant, has decumbent shoots and the yield is very low. In recent years, knowledge of the function of miRNAs in plants has greatly advanced; high-throughput sequencing technology was used to identify *Medicago truncatula* miRNAs. Alfalfa (*Medicago sativa* L.), which is autotetraploid, allogamous and a perennial plant, has been considered as one of the most often grown perennial forage crops worldwide. However, alfalfa varieties developed from different geographic regions have distinct characteristics in FD, and few studies have been performed with the express purpose of identifying and analyzing miRNAs in response to fall dormancy of alfalfa.

In alfalfa, fall dormancy is an adaptive characteristic related to biomass production and winter survival [52], but the physiological, biochemical, and molecular mechanisms causing FD and how this process occurs is still not clear at present. Dormant alfalfa varieties reduce shoot growth, even cease growth and produce short and decumbent shoots after cutting in the autumn, while non-dormant varieties continue normal shoot regrowth and produce tall upright shoots in the autumn. Dormant alfalfa shows fall dormancy morphology in late summer or early autumn, and the shortening day length and falling temperatures in the seasons have been accepted as the environmental factors that induce dormancy in alfalfa [53]. Plant height is the main index indicating fall dormancy in alfalfa [54], and leaf area could also indicate these variations. From the plant height and leaf areas, we can see that Maverick (DM1) and CUF101 (NM2) grow in normal situations in May, Maverick (DS3) might enter fall dormancy in September, while CUF101 (NS4) did not show fall dormancy morphology at this time.

*Medicago truncatula* has decumbent shoots all year round, only for grazing pasture. There are many papers on genome-wide identification of microRNAs from different treatments of *Medicago truncatula*. According to the newest miRNA database (miRBase 20, released in June 2013), *Medicago truncatula* has the greatest amount of precursors and mature miRNAs in plants [6], [55], [56], even more than *Arabidopsis* and rice [57]. However, the functional study level of miRNAs in *Medicago truncatula* is not as high as *Arabidopsis* and rice, only those of highly conserved miRNAs, such as miR156, miR164 and miR172, have been investigated in model plants. Several conserved miRNAs were identified in the genome of *Medicago truncatula*, based on homology to miRNAs present in other plant species, and their expression was confirmed in several organs/tissues. Five of ten conserved miRNAs analyzed here showed differential expression in water deficits as compared to well-watered plants: miR166 was slightly up-regulated in the roots; miR169 was down-regulated in the roots and miR398a/b and miR408 were up-regulated in both shoots and roots. Conversely, miR156, miR160, miR171, miR319 and miR393 expression levels were not affected by water deficits [10].

In *Arabidopsis thaliana*, the predicted targets of miR156 regulated SPL transcription factors, defining an endogenous flowering pathway [58]. Likewise, miR172 regulates AP2-like genes that are involved in controlling floral organ identity in barley, maize, and rice [6], [55], [56]. In addition, miR156 regulates the expression of miR172 via SPL9, which directly promotes the transcription of miR172b [59]. It has been shown that decreases in N-acetylcysteine 1 (NAC1) mRNA levels due to inducible expression of miR164, result in reduction in lateral root emergence in *Arabidopsis thaliana* [60]. In *Medicago truncatula*, both high-throughput sequencing and RT-qPCR showed that miR169 was down-regulated, and the suppression of miR164 expression may contribute to increases in root/shoot ratios under drought stress [61]. The roots of *Medicago truncatula* have the ability to interact with rhizobia to develop nitrogen-fixing root nodules. MiR164 may be the first miRNA found to be involved in nodule formation, which can target MtHAP2-1 encoding a transcription factor of the CCAAT-binding family with an ability to control nodule meristem function. Overexpression of miR164 leads to blockage of nodule development by down-regulating the expression of MtHAP2-1. MiR164 also has the ability to control the transcript levels, as well as the expression patterns, of their targets, suggesting that they might contribute to developmental robustness [48], [62], [63].

Exploring the expression profile of some key miRNAs may be very important for understanding the mechanism of fall dormancy in alfalfa. We compared three sets of different miRNA expressions; i.e., DS3 vs DM1, DS3 vs NM2 and DS3 vs NS4, and each set had 11 up-regulated miRNAs and nine down-regulated miRNAs. Among these miRNAs, conserved miRNAs miR156 and miR164 were up-regulated target genes and miR172 was a down-regulated target gene. It is conceivable that the different expressions of miR172, miR164 and miR156 miRNA families regulate floral repression and influence plant growth. Therefore, we inferred that shortening day-length and falling temperatures in autumn can cause up-regulation of miR164a, miR164d, miR156g-3p, and miR156e. This might trigger SPL transcription factors and NAC1 genes and repress growth of dormant alfalfa in autumn. In addition, the down-regulation of miR172a expression only appeared in dormant alfalfa, which strengthened floral repression and repressed plant growth (four members of miRNAs belonging to the 172miRNA family were identified). Non-dormant alfalfa continues normal growth in the autumn, and the increased expression of miR172a in three other non-dormant situations relieved floral repression and promoted plant growth.

In *Medicago truncatula*, the regulation of genes encoding copper proteins by miR398a/b and miR408 suggests a link between copper homeostasis and *Medicago truncatula* adaptation, and the up-regulation of miR398a/b and miR408 and the clear down-regulation of their respective target genes [10]. In alfalfa, among the predicted up-regulation miRNAs in three sets (DS3 vs DM1, DS3 vs NM2 and DS3 vs NS4), miR5228 expression was at the highest level by high-throughput sequencing and RT-PCR. The predicted functions of miR5228 at the transcriptome level of *Medicago sativa* focused on serine/threonine-protein phosphatase, Tobacco Mosaic Virus (TMV) resistance protein N-like, apoptosis inducing factor homolog A-like, and coatomer subunit beta-1-like (Table S5). Phytochrome acts as a serine kinase and can autophosphorylate itself or transphosphorylate its partner proteins. The exposure of light to plants triggers translocation of the phytochrome from the cytosol to the nucleus where it interacts with several proteins, including transcriptional factors [64]. Alterations in phytochrome A expression disrupt the circadian clock under low light intensities and inhibit the perception of flower-inducing long day conditions in *Arabidopsis thaliana* [65], [66]. Wang *et al.* also demonstrated that the photoperiod effects on phytochrome B and abscisic acid (ABA) in three alfalfa varieties differing in degree of fall dormancy [67]. At the genome level of *Medicago truncatula*, miR5228 predicted function was sentrin-specific protease chromosome5(chr5) in accordance with the transcriptome level of *Medicago sativa*. This result suggests that miR5228 might play an important role in post-transcriptional gene regulation of fall dormancy in alfalfa. Ubiquitination is an enzymatic, post-translational modification (PTM) process in which a ubiquitin protein is attached to a substrate protein. From KEGG pathway analysis, the main factors of fall dormancy include ubiquitin-mediated proteolysis. Ubiquitination is involved in almost all organism activity, such as cell cycle control, reproductive death, gene expression, and the regulation of transcription. In this way, we inferred that increased levels of miR5228 may reduce alfalfa growth during fall dormancy. The current data show that the expression of miR5228 in dormant alfalfa (DS3) is significantly higher than in dormant alfalfa (DM1) and non-dormant alfalfa (NM2, and NS4). These results may explain why organ growth slows down in dormant alfalfa. Based on *de novo* transcriptome sequencing and identification of fall dormancy related genes in alfalfa, serine/threonine-protein phosphatase and ubiquitination were the main factors to target genes regulating fall dormancy functions. For the rest of the predicted up-regulation miRNAs in three sets of alfalfa, miR5279 had no hits, and the other microRNAs (miR2674, miR5299, miR2643a, miR5205b, and miR2655b) had hypothetical proteins, resistance proteins and predicted proteins. For example, miR2643a predicted functions include acting as an IAA-amino acid hydrolase ILR1-like protein, F-box/kelch-repeat protein, F-box family protein, phototropin, and heat shock protein. Later, Fan *et al.* and Du *et al.* found a negative correlation between IAA content and fall dormancy in dormant alfalfa leaves under a short photoperiod and falling temperature [68], [69]. The IAA-amino acid conjugate plays a critical role in plant growth, and it is inferred that increased levels of miR2643a may reduce the content of the IAA-amino acid conjugate and further lead to stopping growth of alfalfa. F-box protein is an expanding family of eukaryotic proteins characterized by an F-box motif, which has specificity of substrate recognition in the ubiquitin-mediated proteolysis. These proteins have been shown to be critical for many physiological processes, such as cell cycle transition, signal transduction, and development [70], [71]. Phototropin is one kind of photoreceptor whose wavebands of the solar spectrum are 320–500 nm. It belongs to serine/threonine kinase with flavin as a chromophore and it has phototropism. Fall dormancy is affected by the photoperiod and is further influenced by phototropin. The miR2674 function annotation is resistance protein, and this was consistent with fall dormancy being a kind of biological regulation of response to stress from the analysis of GO enrichment and KEGG pathways.

In *Medicago truncatula*, ethylene-responsive miRNAs in roots have been identified by high-throughput sequencing. Eight miRNAs were shown to be down-regulated after exposure to ethylene, and the potential role of these miRNAs in the ethylene-induced inhibition of root elongation was discussed [72]. Among the predicted down-regulation of miRNAs in three sets of alfalfa, miR52671 and miR2629h had no hits, and only predicted a common function as a kind of hypothetical protein. MiR2676a can function as the mediator of an RNA polymerase II transcription subunit, F-box/WD-40 repeat-containing protein chr5, small ubiquitin-related modifier, and cyclin-like F-box calcium-binding mitochondrial carrier protein. MiR2593e can react on NAC-domain protein chr3, MiR5286b, miR5286a and miR2634, have similar functions with some up-regulating miRNAs in the regulation of biological processes of alfalfa.

MiRNA study in *Medicago truncatula* is advancing quickly, however, miRNA study in *Medicago truncatula* is not as detailed in contrast to *Arabidopsis* and rice. Because most miRNAs have been identified and finding novel miRNAs from *Medicago truncatula* is increasingly difficult, researchers are beginning to pay more attention to the functional study of important miRNAs in diverse biochemical and physiological processes, which will become the trend of miRNA study in *Medicago truncatula* [57]. A more effective approach to gene silencing has been to produce sRNAs in alfalfa. Verdonk and Sullivan indicated that amiRNA silencing constructs of miR391a can be functional in alfalfa to specifically silence gene expression [33].

Some special miRNAs were found to be involved in fall dormancy of alfalfa, based on the analysis of GO classification and the KEGG pathway. Fall dormancy strongly correlates to the level of alfalfa yields. The growth of fall dormant alfalfa varieties often surpasses those of non-fall dormant alfalfa varieties in winter, but enters dormancy much earlier than non-fall dormant varieties. In this study, some novel miRNAs candidates were found to be specific to dormant alfalfa and non-dormant alfalfa. These novel miRNA candidates included novel_1, novel_3, novel_42, novel_45, novel_17, novel_75, and novel_76, whose expression levels were both high and significantly different in three sets, five of which were confirmed by Northern blotting analysis. It has been suggested that these miRNAs may play a vital role in response to fall dormancy in alfalfa. However, their functions are not yet fully understood and need further study.

## Conclusions

In conclusion, four sRNA libraries were generated and sequenced from alfalfa leaves in two standard varieties, at two time points, using high-throughput sequencing technology. Our study generated 7,536,934, 6,572,924, 6,548,094, and 6,627,260 clean reads, and identified 566 and 583 potential miRNAs related to fall dormancy in standard variety (Maverick and CUF101) at the genome level of *Medicago truncatula* and transcriptome of *Medicago sativa*. Based on the analysis between GO classification and the KEGG pathway, we inferred that the regulation mechanism of fall dormancy was biological regulation of responses to stress in the metabolic pathway. We also identified 28miRNAs associated with fall dormancy in standard variety (Maverick and CUF101), including 20 known miRNAs and eight novel miRNA candidates. The known fall dormancy-responsive miRNAs were found to be involved in diverse cellular processes in plants, including development, flowering, dormancy, transcription, protein degradation, cell cycle transition, signal transduction, and adaptation. These miRNA-mediated networks could play crucial roles during the dormancy of alfalfa, and our miRNA data provides valuable information regarding further functional analysis of miRNAs involved in fall dormancy in alfalfa.

## Supporting Information

Figure S1 **Sampling stage in May and September of two different standard alfalfa varieties (Maverick and CUF101).**
(TIF)

Figure S2 **Error distribution of position along reads generated from four samples.**
(TIF)

Figure S3 **GC content distribution and miRNA first nucleotide bias generated from four samples.**
(TIF)

Figure S4 **Log_2_ (Fold Change) of differential miRNA expression of DS3 vs DM1, DS3 vs NM2 DS3, and DS3 vs NS4.**
(TIF)

Figure S5 **Cluster analysis of differentially expressed sRNAs in different types of dormant alfalfa.** Clustering was performed based on fold-changes between DM1, NM2, DS3, and NS4. 146miRNAs were potentially involved in the regulation of fall dormancy.
(TIF)

Table S1 **Primer sequences used for qRT-PCR.**
(XLS)

Table S2 **Novel miRNAs identified in different types of dormant alfalfa, listed sequences and abundances for novel miRNAs were identified.**
(XLS)

Table S3 **MiRNA family analysis results of novel miRNAs and known miRNAs.**
(XLS)

Table S4 **The intersection of the two predicting tools for potential miRNAs related to fall dormancy at the genome level of** ***Medicago truncatula.***
(XLS)

Table S5 **The intersection of the two predicting tools for potential miRNAs related to fall dormancy at the transcriptome level of** ***Medicago sativa.***
(XLS)

Table S6 **The statistical enrichment of differential expressions of miRNA target gene candidates in KEGG pathways.**
(XLS)

Table S7 **Comparison between DS3 and DM1 of the normalized expressions of differential miRNAs.**
(XLS)

Table S8 **Comparison between DS3 and NM2 of the normalized expressions of differential miRNAs.**
(XLS)

Table S9 **Comparison between DS3 and NS4 of the normalized expressions of differential miRNAs.**
(XLS)

Table S10 **New differential miRNA responses to fall dormancy in different types of dormant alfalfa.**
(XLS)

## References

[1] Khraiwesh B, Arif MA, Seumel GI, Ossowski S, Weigel D, et al (2010) Transcriptional control of gene expression by microRNAs. Cell 140:111–122. 20085706 10.1016/j.cell.2009.12.023

[2] Jones-Rhoades MW, Bartel DP, Bartel B (2006) MicroRNAs and their regulatory roles in plants. Annu Rev Plant Biol. 19–53. 10.1146/annurev.arplant.57.032905.105218 16669754

[3] Lee RC, Feinbaum RL, Ambros V (1993) The C. elegans heterochronic gene lin-4 encodes small RNAs with antisense complementarity to lin-14. Cell 75:843–854. 8252621 10.1016/0092-8674(93)90529-y

[4] Mallory AC, Vaucheret H (2006) Functions of microRNAs and related small RNAs in plants. Nature Genetics 38:S31–S36. 16736022 10.1038/ng1791

[5] Chen X (2004) A microRNA as a translational repressor of APETALA2 in Arabidopsis flower development. Science 303:2022–2025. 12893888 10.1126/science.1088060 PMC5127708

[6] Aukerman MJ, Sakai H (2003) Regulation of flowering time and floral organ identity by a microRNA and its APETALA2-like target genes. The Plant Cell Online 15:2730–2741. 10.1105/tpc.016238 PMC280575 14555699

[7] Wang JW, Wang LJ, Mao YB, Cai WJ, Xue HW, et al (2005) Control of root cap formation by microRNA-targeted auxin response factors in Arabidopsis. The Plant cell online 17:2204–2216. 10.1105/tpc.105.033076 PMC1182483 16006581

[8] Zhou ZS, Zeng HQ, Liu ZP, Yang ZM (2012) Genome–wide identification of Medicago truncatula microRNAs and their targets reveals their differential regulation by heavy metal. Plant, cell & environment 35:86–99. 10.1111/j.1365-3040.2011.02418.x 21895696

[9] Zhou ZS, Huang SQ, Yang ZM (2008) Bioinformatic identification and expression analysis of new microRNAs from Medicago truncatula Biochemical and biophysical research communications. 374:538–542. 10.1016/j.bbrc.2008.07.083 18662674

[10] Trindade I, Capitão C, Dalmay T, Fevereiro MP, dos Santos DM (2010) miR398 and miR408 are up-regulated in response to water deficit in Medicago truncatula. Planta 231:705–716. 20012085 10.1007/s00425-009-1078-0

[11] Zhang J, Xu Y, Huan Q, Chong K (2009) Deep sequencing of Brachypodium small RNAs at the global genome level identifies microRNAs involved in cold stress response. BMC genomics 10:449. 19772667 10.1186/1471-2164-10-449 PMC2759970

[12] Jones-Rhoades MW, Bartel DP (2004) Computational identification of plant microRNAs and their targets, including a stress-induced miRNA. Molecular cell 14:787–799. 15200956 10.1016/j.molcel.2004.05.027

[13] Dien BS, Jung HJG, Vogel KP, Casler MD, Lamb JF, et al (2006) Chemical composition and response to dilute-acid pretreatment and enzymatic saccharification of alfalfa, reed canarygrass, and switchgrass. Biomass and Bioenergy 30:880–891.

[14] Samac DA, Jung H, Lamb J (2006) Development of alfalfa (Medicago sativa L.) as a feedstock for production of ethanol and other bioproducts. Chemical Industries-New York-Marcel Dekker- 112:79.

[15] Trinh T, Ratet P, Kondorosi E, Durand P, Kamaté K, et al (1998) Rapid and efficient transformation of diploid Medicago truncatula and Medicago sativa ssp. falcata lines improved in somatic embryogenesis. Plant Cell Reports 17:345–355. 10.1007/s002990050405 30736570

[16] Wang C, Ma B, Yan X, Han J, Guo Y, et al (2009) Yields of alfalfa varieties with different fall-dormancy levels in a temperate environment. Agronomy Journal 101:1146–1152.

[17] Smith D (1961) Association of fall growth habit and winter survival in alfalfa. Canadian Journal of Plant Science 41:244–251.

[18] Larson KL, Smith D (1963) Association of various morphological characters and seed germination with the winterhardiness of alfalfa. Crop Science 3:234–237.

[19] Stout DG, Hall JW (1989) Fall growth and winter survival of alfalfa in interior British Columbia. Canadian Journal of Plant Science 69:491–499.

[20] Schwab P, Barnes D, Sheaffer C (1996) The relationship between field winter injury and fall growth score for 251 alfalfa cultivars. Crop Science 36:418–426.

[21] Larson K, Smith D (1963) Association of various morphological characters and seed germination with the winterhardiness of alfalfa. Crop Science 3:234–237.

[22] Bula R, Smith D (1954) Cold resistance and chemical composition in overwintering alfalfa, red clover, and sweetclover. Agronomy Journal 46:397–401.

[23] Volenec JJ, Boyce PJ, Hendershot KL (1991) Carbohydrate metabolism in taproots of *Medicago sativa* L. during winter adaptation and spring regrowth. Plant Physiology 96:786–793. 16668255 10.1104/pp.96.3.786 PMC1080844

[24] Castonguay Y, Nadeau P, Lechasseur P, Chouinard L (1995) Differential accumulation of carbohydrates in alfalfa cultivars of contrasting winterhardiness. Crop Science 35:509–516.

[25] Cunningham SM, Volenec JJ (1998) Seasonal carbohydrate and nitrogen metabolism in roots of contrasting alfalfa (*Medicago sativa* L.) cultivars. Journal of Plant Physiology 153:220–225.

[26] Cunningham S, Gana J, Volenec J, Teuber L (2001) Winter hardiness, root physiology, and gene expression in successive fall dormancy selections from ‘Mesilla’ and ‘CUF 101’ alfalfa. Crop Science 41:1091–1098.

[27] Heinrichs DH, Troelsen JE, Clark KW (1960) Winter hardiness evaluation in alfalfa. Canadian Journal of Plant Science 40:638–644.

[28] Wang C, Ma BL, Han J, Wang Y, Gao Y, et al (2008) Photoperiod effect on phytochrome and abscisic acid in alfalfa varieties differing in fall dormancy. Journal of Plant Nutrition 31:1257–1269.

[29] Ariss JJ, Vandemark GJ (2007) Assessment of genetic diversity among nondormant and semidormant alfalfa populations using sequence-related amplified polymorphisms. Crop science 47:2274–2284.

[30] Fairey D, Fairey N, Lefkovitch L (1996) The relationship between fall dormancy and germplasm source in North American alfalfa cultivars. Canadian journal of plant science 76:429–433.

[31] Szittya G, Moxon S, Santos DM, Jing R, Fevereiro MP, et al (2008) High-throughput sequencing of Medicago truncatula short RNAs identifies eight new miRNA families. BMC genomics 9:593. 19068109 10.1186/1471-2164-9-593 PMC2621214

[32] Lelandais-Brière C, Naya L, Sallet E, Calenge F, Frugier F, et al (2009) Genome-wide Medicago truncatula small RNA analysis revealed novel microRNAs and isoforms differentially regulated in roots and nodules. The Plant Cell Online 21:2780–2796. 10.1105/tpc.109.068130 PMC2768930 19767456

[33] Verdonk JC, Sullivan ML (2012) Artificial microRNA (amiRNA) induced gene silencing in alfalfa (Medicago sativa). Botany 91:117–122.

[34] Teuber L, Taggard K, Gibbs L, McCaslin M, Peterson M, et al**.** (1998) Fall dormancy; 2–6.

[35] Wen M, Shen Y, Shi S, Tang T (2012) miREvo: an integrative microRNA evolutionary analysis platform for next-generation sequencing experiments. BMC bioinformatics 13:140. 22720726 10.1186/1471-2105-13-140 PMC3410788

[36] Friedländer MR, Mackowiak SD, Li N, Chen W, Rajewsky N (2012) miRDeep2 accurately identifies known and hundreds of novel microRNA genes in seven animal clades. Nucleic acids research 40:37–52. 21911355 10.1093/nar/gkr688 PMC3245920

[37] Langmead B, Trapnell C, Pop M, Salzberg SL (2009) Ultrafast and memory-efficient alignment of short DNA sequences to the human genome. Genome Biol 10:R25. 19261174 10.1186/gb-2009-10-3-r25 PMC2690996

[38] Li R, Li Y, Kristiansen K, Wang J (2008) SOAP: short oligonucleotide alignment program. Bioinformatics 24:713–714. 18227114 10.1093/bioinformatics/btn025

[39] Zhou L, Chen J, Li Z, Li X, Hu X, et al (2010) Integrated profiling of microRNAs and mRNAs: microRNAs located on Xq27. 3 associate with clear cell renal cell carcinoma. PloS one 5:e15224. 21253009 10.1371/journal.pone.0015224 PMC3013074

[40] Storey JD (2003) The positive false discovery rate: A Bayesian interpretation and the q-value. Annals of statistics: 2013–2035.

[41] Moxon S, Schwach F, Dalmay T, MacLean D, Studholme DJ, et al (2008) A toolkit for analysing large-scale plant small RNA datasets. Bioinformatics 24:2252–2253. 18713789 10.1093/bioinformatics/btn428

[42] Arocho A, Chen B, Ladanyi M, Pan Q (2006) Validation of the 2−ΔΔCt calculation as an alternate method of data analysis for quantitative PCR of BCR-ABL P210 transcripts. Diagnostic Molecular Pathology 15:56–61. 16531770 10.1097/00019606-200603000-00009

[43] Deng Y, Wu S, Zhou H, Bi X, Wang Y, et al (2013) Effects of a miR-31, Runx2, and Satb2 regulatory loop on the osteogenic differentiation of bone mesenchymal stem cells. Stem cells and development 22:2278–2286. 23517179 10.1089/scd.2012.0686

[44] Lacombe S, Nagasaki H, Santi C, Duval D, Piégu B, et al (2008) Identification of precursor transcripts for 6 novel miRNAs expands the diversity on the genomic organisation and expression of miRNA genes in rice. BMC plant biology 8:123. 19055717 10.1186/1471-2229-8-123 PMC2607281

[45] Kim SW, Li Z, Moore PS, Monaghan AP, Chang Y, et al**.** (2010) A sensitive non-radioactive northern blot method to detect small RNAs. Nucleic acids research: gkp1235. 10.1093/nar/gkp1235 PMC2853138 20081203

[46] Yifen C, Zhizhong C, Shangli S. TianYoufeng (1990) A modified technique measuring leaf area of alfalfa. Pratacultural Science 7:60–62.

[47] Meyers BC, Axtell MJ, Bartel B, Bartel DP, Baulcombe D, et al (2008) Criteria for annotation of plant MicroRNAs. The Plant Cell Online 20:3186–3190. 10.1105/tpc.108.064311 PMC2630443 19074682

[48] Ambros V, Bartel B, Bartel DP, Burge CB, Carrington JC, et al (2003) A uniform system for microRNA annotation. Rna 9:277–279. 12592000 10.1261/rna.2183803 PMC1370393

[49] Mao X, Cai T, Olyarchuk JG, Wei L (2005) Automated genome annotation and pathway identification using the KEGG Orthology (KO) as a controlled vocabulary. Bioinformatics 21:3787–3793. 15817693 10.1093/bioinformatics/bti430

[50] Zhou L, Liu Y, Liu Z, Kong D, Duan M, et al (2010) Genome-wide identification and analysis of drought-responsive microRNAs in Oryza sativa. Journal of experimental botany 61:4157–4168. 20729483 10.1093/jxb/erq237

[51] Zhao B, Ge L, Liang R, Li W, Ruan K, et al (2009) Members of miR-169 family are induced by high salinity and transiently inhibit the NF-YA transcription factor. BMC Molecular Biology 10:29. 19351418 10.1186/1471-2199-10-29 PMC2670843

[52] Rimi F, Macolino S, Leinauer B, Lauriault LM, Ziliotto U (2014) Fall Dormancy and Harvest Stage Impact on Alfalfa Persistence in a Subtropical Climate. Agronomy Journal.

[53] McKenzie J, Paquin R, Duke SH (1988) Cold and heat tolerance. Alfalfa and alfalfa improvement: 259–302.

[54] Schneider M, Foord K, Teuber L (1984) Unifoliate internode length of alfalfa: Relationship to origin and fall dormancy. p. Agronomy Abstracts ASA, Madison, WI Unifoliate internode length of alfalfa: Relationship to origin and fall dormancy: 96.

[55] Lauter N, Kampani A, Carlson S, Goebel M, Moose SP (2005) microRNA172 down-regulates glossy15 to promote vegetative phase change in maize. Proceedings of the National Academy of Sciences of the United States of America 102:9412–9417. 15958531 10.1073/pnas.0503927102 PMC1166634

[56] Nair SK, Wang N, Turuspekov Y, Pourkheirandish M, Sinsuwongwat S, et al (2010) Cleistogamous flowering in barley arises from the suppression of microRNA-guided HvAP2 mRNA cleavage. Proceedings of the National Academy of Sciences 107:490–495. 10.1073/pnas.0909097107 PMC2806734 20018663

[57] Wang TZ (2014) Recent Research Progress on MicroRNAs in Medicago truncatula. Plant Cell 18:2051–2065.

[58] Wang JW, Czech B, Weigel D (2009) miR156-Regulated SPL Transcription Factors Define an Endogenous Flowering Pathway in Arabidopsis thaliana. Cell 138:738–749. 19703399 10.1016/j.cell.2009.06.014

[59] Wu G, Park MY, Conway SR, Wang JW, Weigel D, et al (2009) The Sequential Action of miR156 and miR172 Regulates Developmental Timing in Arabidopsis. Cell 138:750–759. 19703400 10.1016/j.cell.2009.06.031 PMC2732587

[60] Guo HS, Xie Q, Fei JF, Chua NH (2005) MicroRNA directs mRNA cleavage of the transcription factor NAC1 to downregulate auxin signals for Arabidopsis lateral root development. The Plant Cell Online 17:1376–1386. 10.1105/tpc.105.030841 PMC1091761 15829603

[61] Wang T, Chen L, Zhao M, Tian Q, Zhang WH (2011) Identification of drought-responsive microRNAs in Medicago truncatula by genome-wide high-throughput sequencing. BMC genomics 12:367. 21762498 10.1186/1471-2164-12-367 PMC3160423

[62] Sieber P, Wellmer F, Gheyselinck J, Riechmann JL, Meyerowitz EM (2007) Redundancy and specialization among plant microRNAs: role of the MIR164 family in developmental robustness. Development 134:1051–1060. 17287247 10.1242/dev.02817

[63] Combier JP, Frugier F, de Billy F, Boualem A, El-Yahyaoui F, et al (2006) MtHAP2-1 is a key transcriptional regulator of symbiotic nodule development regulated by microRNA169 in Medicago truncatula. Genes & development 20:3084–3088. 17114582 10.1101/gad.402806 PMC1635144

[64] Sharma R (2001) Phytochrome: A serine kinase illuminates the nucleus! Current Science-Bangalore-. 80:178–188.

[65] Reed JW, Nagatani A, Elich TD, Fagan M, Chory J (1994) Phytochrome A and phytochrome B have overlapping but distinct functions in Arabidopsis development. Plant Physiology 104:1139–1149. 12232154 10.1104/pp.104.4.1139 PMC159274

[66] Somers DE, Devlin PF, Kay SA (1998) Phytochromes and cryptochromes in the entrainment of the Arabidopsis circadian clock. Science 282:1488–1490. 9822379 10.1126/science.282.5393.1488

[67] Wang C, Ma B, Han J, Wang Y, Gao Y, et al (2008) Photoperiod effect on phytochrome and abscisic acid in alfalfa varieties differing in fall dormancy. Journal of Plant Nutrition 31:1257–1269.

[68] Fan W, Sun X, Du H, Yan X, Shi Y, et al (2014) Photoperiod Effect on Phytochromes and Endogenous Hormones of Alfalfa with Different Fall-dormancy Acta Prataculturae Sinica. 23:177–184.

[69] Du H, Liang M, Fan W, Shi Y, Wang C (2013) The effect of phytochrome and Endogenous Hormones on fall dormancy of differnt alfalfa in temperature. Acta Agrestia Sinica 21:708–713.

[70] Ciechanover A (1998) The ubiquitin–proteasome pathway: on protein death and cell life. The EMBO journal 17:7151–7160. 9857172 10.1093/emboj/17.24.7151 PMC1171061

[71] Hart M, Concordet J, Lassot I, Albert I, Del los Santos R, et al (1999) The F-box protein β-TrCP associates with phosphorylated β-catenin and regulates its activity in the cell. Current biology 9:207–211. 10074433 10.1016/s0960-9822(99)80091-8

[72] Chen L, Wang T, Zhao M, Zhang W (2012) Ethylene-responsive miRNAs in roots of Medicago truncatula identified by high-throughput sequencing at whole genome level. Plant Science 184:14–19. 22284705 10.1016/j.plantsci.2011.11.007

