# Supplementary figures and images for: Genome-Wide Identification of Different Dormant Medicago sativa L. MicroRNAs in Response to Fall Dormancy
Source: PLoS One. 2014 Dec 4;9(12):e114612. doi: 10.1371/journal.pone.0114612 (PMC4256440; doi:10.1371/journal.pone.0114612)

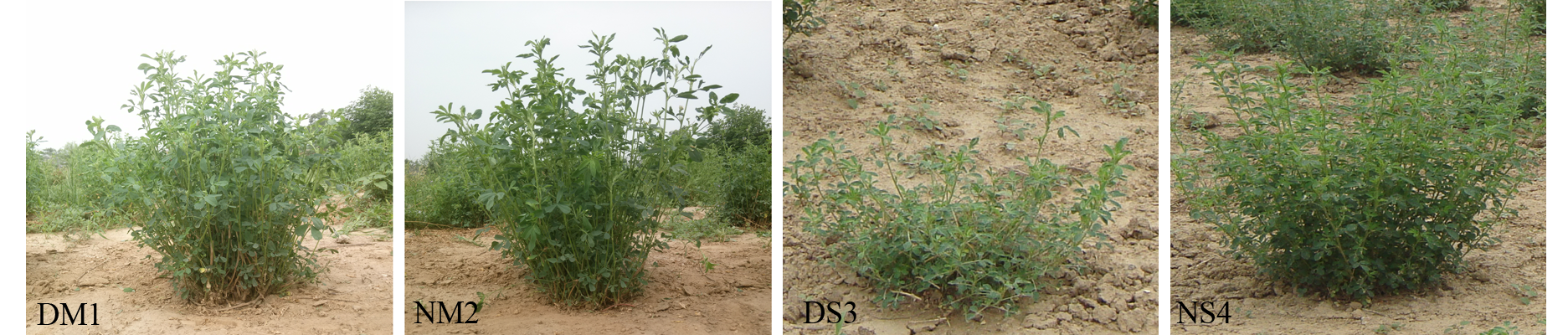

Supplement: Figure S1 — Sampling stage in May and September of two different standard alfalfa varieties (Maverick and CUF101). (TIF) [file pone.0114612.s001.tif]

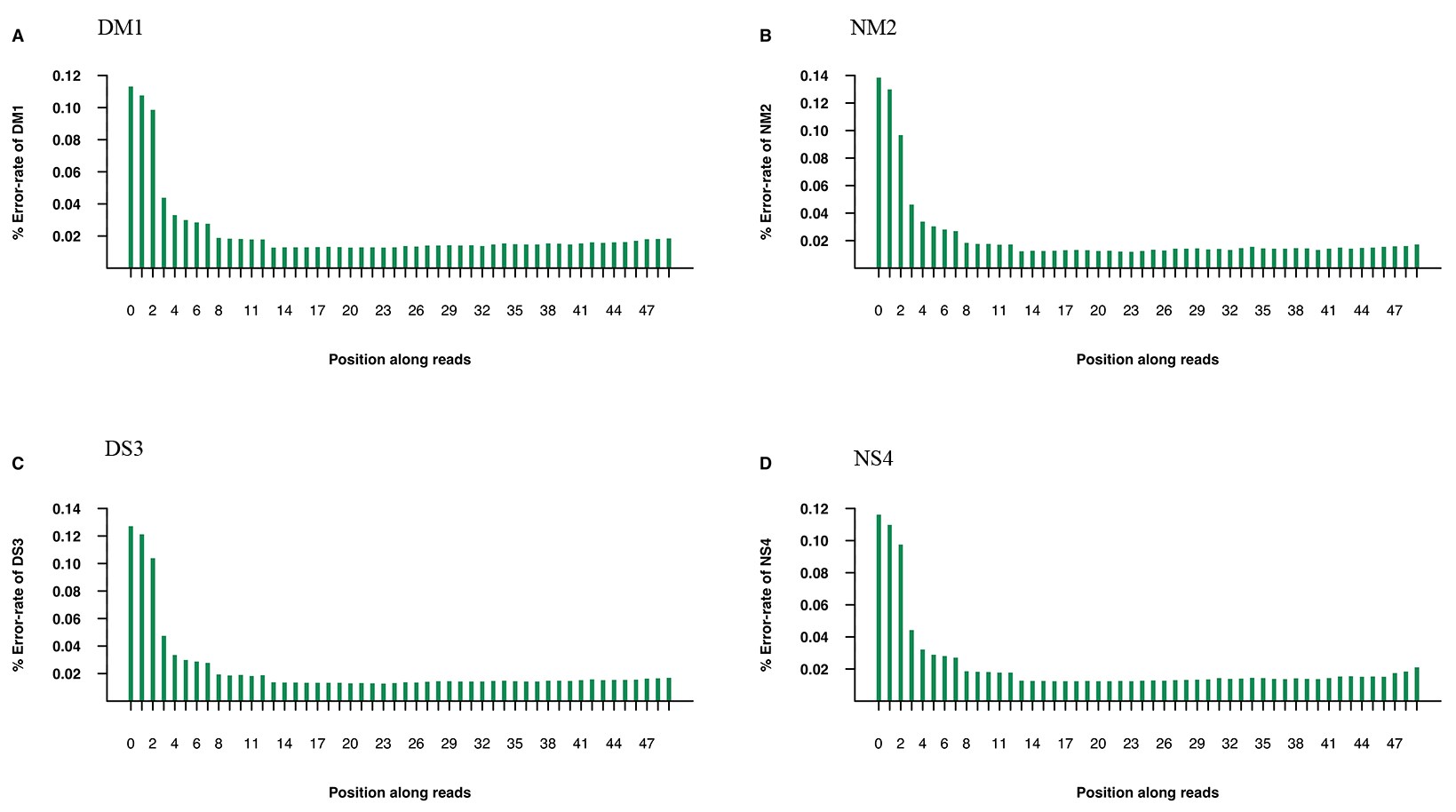

Supplement: Figure S2 — Error distribution of position along reads generated from four samples. (TIF) [file pone.0114612.s002.tif]

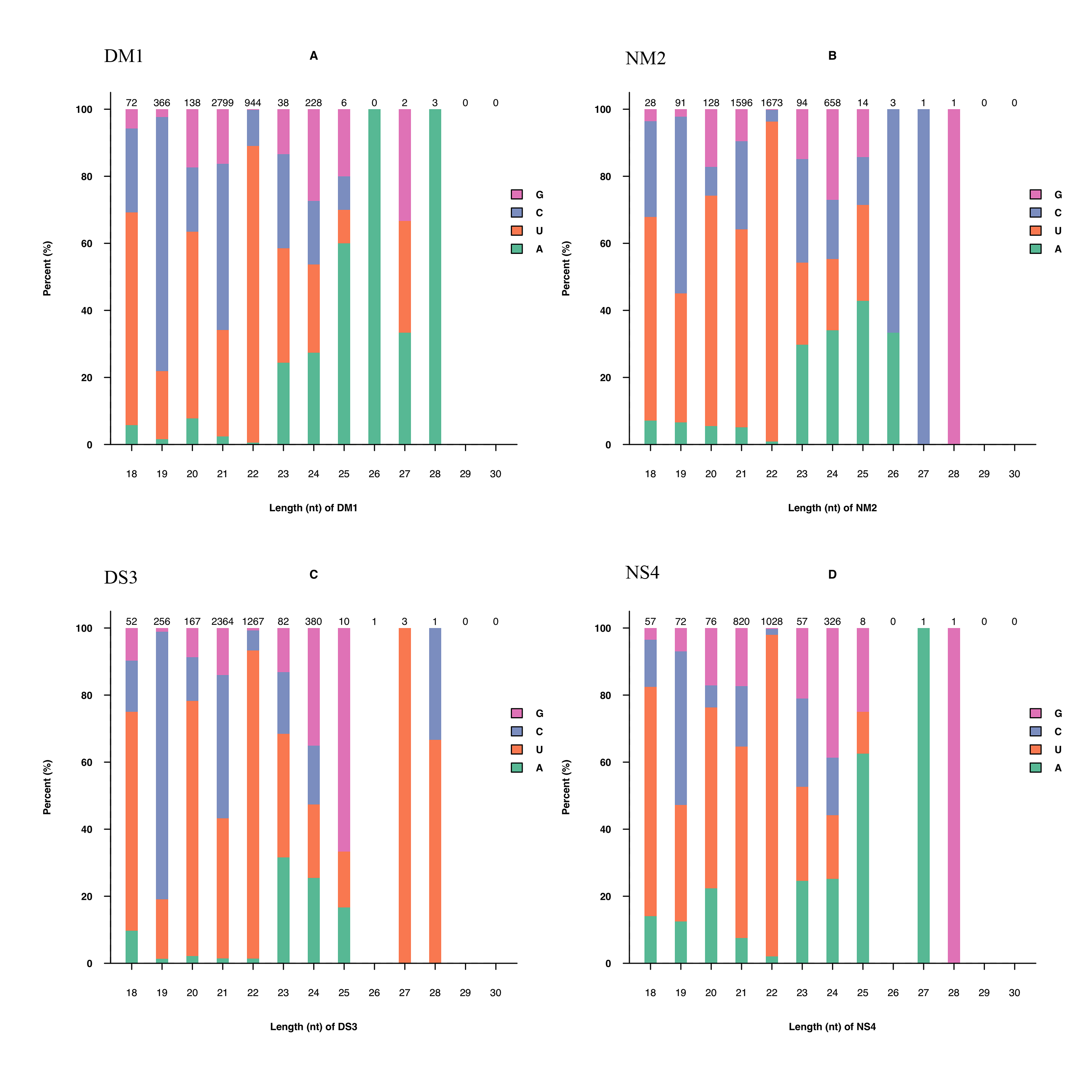

Supplement: Figure S3 — GC content distribution and miRNA first nucleotide bias generated from four samples. (TIF) [file pone.0114612.s003.tif]

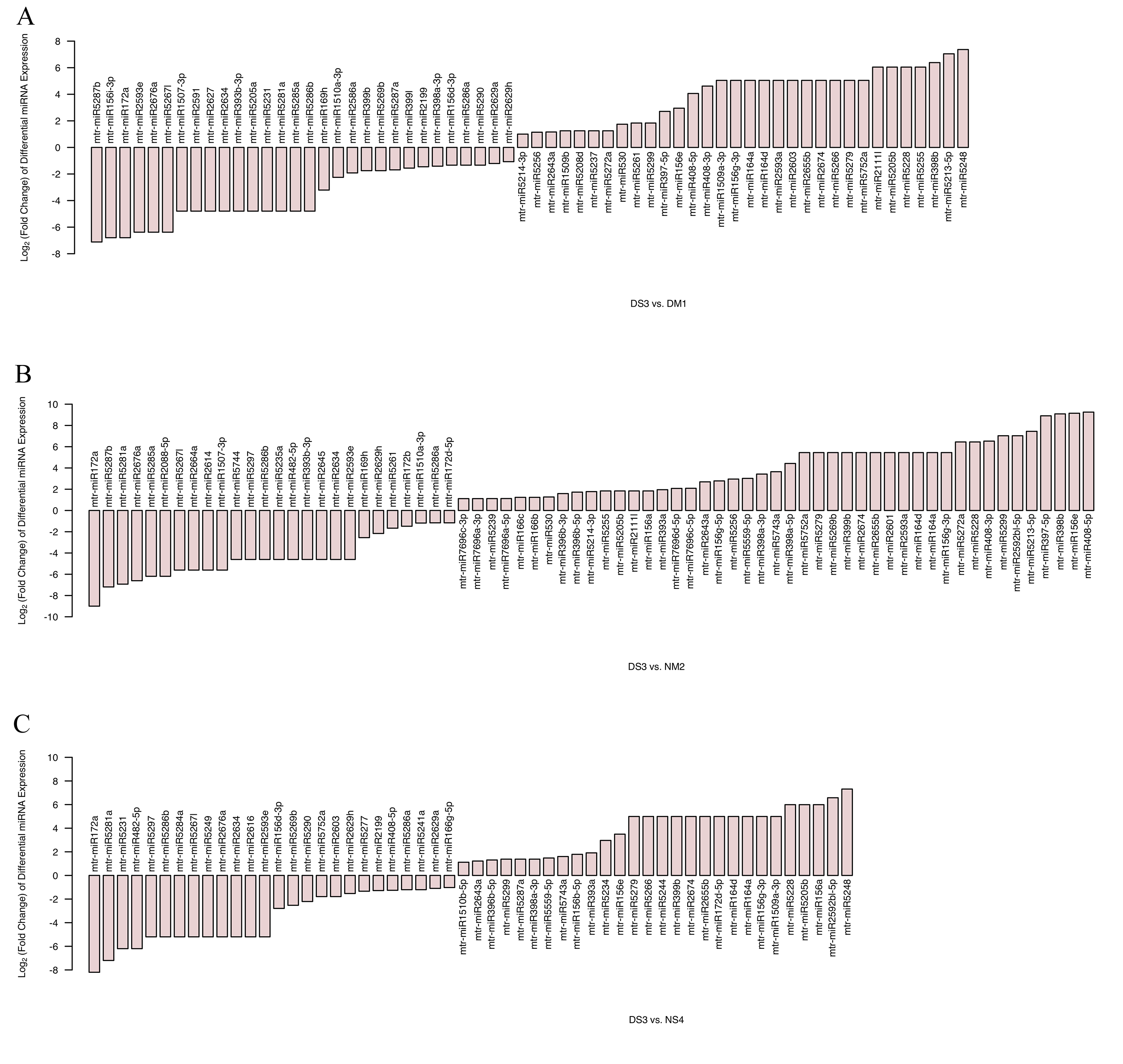

Supplement: Figure S4 — Log2 (Fold Change) of differential miRNA expression of DS3 vs DM1, DS3 vs NM2 DS3, and DS3 vs NS4. (TIF) [file pone.0114612.s004.tif]

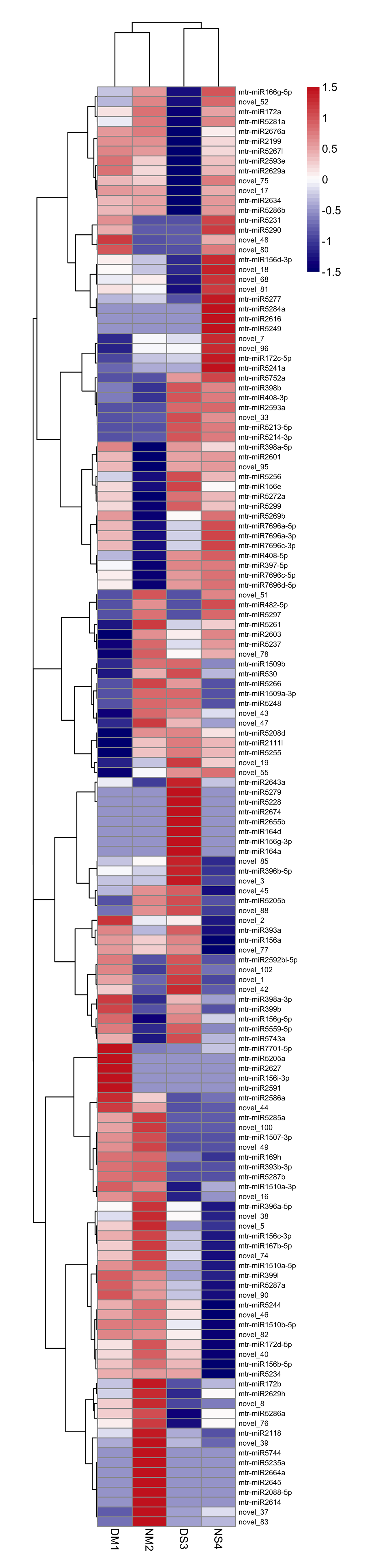

Supplement: Figure S5 — Cluster analysis of differentially expressed sRNAs in different types of dormant alfalfa. Clustering was performed based on fold-changes between DM1, NM2, DS3, and NS4. 146miRNAs were potentially involved in the regulation of fall dormancy. (TIF) [file pone.0114612.s005.tif]
